# Stimulus-Entrained Oscillatory Activity Propagates as Waves from Area 18 to 17 in Cat Visual Cortex

**DOI:** 10.1371/journal.pone.0041960

**Published:** 2012-07-25

**Authors:** Lian Zheng, Haishan Yao

**Affiliations:** 1 Institute of Neuroscience and State Key Laboratory of Neuroscience, Shanghai Institutes for Biological Sciences, Chinese Academy of Sciences, Shanghai, China; 2 Graduate School of Chinese Academy of Sciences, Shanghai, China; University of Southern California, United States of America

## Abstract

Previous studies in cat visual cortex reported that area 18 can actively drive neurons in area 17 through cortico-cortical projections. However, the dynamics of such cortico-cortical interaction remains unclear. Here we used multielectrode arrays to examine the spatiotemporal pattern of neuronal activity in cat visual cortex across the 17/18 border. We found that full-field contrast reversal gratings evoked oscillatory wave activity propagating from area 18 to 17. The wave direction was independent of the grating orientation, and could not be accounted for by the spatial distribution of receptive field latencies, suggesting that the waves are largely mediated by intrinsic connections in the cortex. Different from the evoked waves, spontaneous waves propagated along both directions across the 17/18 border. Together, our results suggest that visual stimulation may enhance the flow of information from area 18 to 17.

## Introduction

Theoretical studies showed that a traveling wave is an emergent behavior of systems with spatially restricted connectivity [1], [2], [3]. Experimental studies have observed waves in a variety of brain regions, including the olfactory bulb [4], [5], [6] , hippocampus [7], primary sensory cortices [8], [9], [10], [11], [12], [13], [14], [15], [16], and motor cortex [17], suggesting that waves may contribute to cortical function.

In the visual cortex, various wave-like activities have been observed. For example, a single spike can initiate a radial wave across the surface of cortex [18], and a local visual stimulus can evoke a traveling wave spreading from the retinotoptic representation of the stimulus to neighboring cortical area [8], [10]. The biophysical substrate for the spreading activity may involve horizontal connections in the visual cortex [19]. Traveling waves were also reported in the visual cortices of ferrets and turtles [11], [20], [21]. These waves traveled across the border of different visual areas, probably mediated by long range cortico-cortical connections. The inter-cortical wave-like activity may serve as a means of communication between different areas [11], [22].

Connections between areas 17 and 18 in cat visual cortex are spatially reciprocal [23], [24], [25]. Previous studies showed that neurons in area 17 can be directly driven by neurons in area 18 in addition to the feedforward inputs from layer 4 [26], [27], suggesting that visual information may flow from area 18 to 17. At the population level, however, the spatiotemporal pattern of dynamic interaction between the two areas remains to be investigated. In particular, it is not clear whether the information flow can be manifested as propagating waves. On the other hand, since waves may occur under both sensory stimulation and spontaneous conditions [9], [28], it is also of interest to examine the relationship between the dynamics of evoked and spontaneous activities.

In this study, we have examined the spatiotemporal properties of population activity across areas 17 and 18 by recording local field potentials (LFPs) from the superficial layers with multielectrode arrays. Using full-field contrast reversal gratings that covered the receptive fields (RFs) of all recording sites, we showed that the visually evoked LFPs exhibited systematic phase shifts across the cortical surface, and the activity propagated as waves from area 18 to 17. The direction of the waves was independent of stimulus orientation and the direction of phase gradient could not be accounted for by the spatial gradient of RF latencies. Furthermore, the wave speed increased with the frequency of neuronal oscillation. In spontaneous activity, propagating waves traveled in both directions between areas 17 and 18. Thus, visual stimulation may modulate spontaneous activity to facilitate wave propagation from area 18 to 17.

## Materials and Methods

### Surgery

All procedures were in accordance with National Institutes of Health Guidelines, and the protocol was approved by the Biological Research Ethics Committee of the Shanghai Institutes for Biological Sciences, Chinese Academy of Sciences (permit number: ER-SIBS-221105C).

The methods for surgery have been previously described in detail [29]. Briefly, adult cats (weighing 1.5–4.5 kg each) were initially anesthetized with ketamine (25–30 mg/kg, intramuscularly) and injected with atropine sulfate (0.05 mg/kg, subcutaneously). After tracheotomy, the animals were artificially ventilated. During recordings, anesthesia was maintained with sodium pentothal (3 mg/kg/h) or urethane (13–20 mg/kg/h) and paralysis with Gallamine (10–20 mg/kg/h). The end-tidal CO_2_ was maintained at ∼3.5%, and body temperature at 37.5°C–38.5°C. Eyes were fitted with appropriate contact lenses with artificial pupils of 3-mm diameter, and focused on a tangent screen. A craniotomy (∼6 mm in diameter) was performed over the primary visual cortex (centered at P2–4 and L2–3, including both areas 17 and 18).

### Recordings

Recordings were made with multielectrode arrays (Blackrock Microsystems), consisting of 8×8 or 10×10 grids of microelectrodes (1-mm electrode length, 400-µm electrode separation). To record from the superficial layers, the arrays were inserted 0.5–0.6 mm into the cortex using a pneumatic insertion device. To prevent pulsation, the array and the exposed cortex were covered in 1.5–2% agar. Signals were amplified using a Cerebus 128-channel system (Blackrock microsystems). All LFP signals were sampled at 2 KHz per channel with a wide-band front-end filter (0.3–500 Hz). LFP data were post-processed by removing channels that may not be functional due to broken electrodes or noise.

### Visual Stimulation

Visual stimuli were generated with a PC containing a Leadtek GeForce 6800 video card and displayed on a CRT monitor (Sony CPD-G520, mean luminance of 32 cd/m^2^, 1024×768 resolution, refresh rate 120 Hz) placed 57 cm away from the animal's eyes. Luminance nonlinearities were corrected through software. Stimuli were presented to the contralateral eye. For each experiment, we first mapped the receptive fields (RFs) of the LFP responses using sparse noise stimuli, in which a white or black square (0.5°–2°) was flashed on a gray background at each of the 16×16 (or 12×12) positions in a pseudorandom sequence. The stimuli were presented with an effective frame rate of 60 Hz so that each sparse noise image appeared for 16.7 ms. Each square was presented 30–60 times. We then presented full-field (20°–30°) contrast reversal gratings (spatial frequency: 0.2–0.5 cycle/°, mostly 0.32 cycle/°; contrast: 75%), covering the RFs of all recording sites. In 10 experiments, the gratings were contrast-reversed at 4 Hz. In 4 experiments, the gratings were contrast-reversed at 2, 3, 4, or 5 Hz. These frequencies were chosen because the optimal temporal frequency for neurons in the primary visual cortex was 3–4 Hz [30], [31]. The gratings were presented at 4 different phases (0°, 90°, 180°, and 270°) and 8 different orientations (spaced at 22.5°). Both the phase and the orientation of the gratings were randomized. Each grating was presented for 1 sec and repeated 80 times. In 9 experiments, we measured spontaneous responses using a blank screen of mean luminance as well as evoked responses using contrast-reversal gratings at 4 Hz. In 4 experiments, we measured spontaneous responses as well as evoked responses using contrast-reversal gratings at 2, 3, 4, and 5 Hz.

### Data Analysis

All data analyses were implemented with custom software written in Matlab.

#### Power Spectrum Estimation

We used a multi-taper method [32] to estimate the power spectrum of the responses. For a given LFP signal 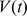, the power spectrum was given by
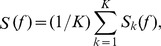


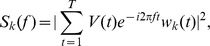
where 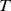 is the length of the LFP signal, and 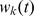 is the 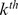 Slepian function. The Slepian functions are orthogonal basis functions that are characterized by bandwidth 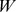 in frequency and length 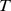 in time. There are 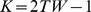 tapers that are spectrally concentrated in the frequency band 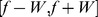. In our analysis, we used 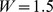 Hz and 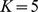.

#### Spatial Coherence

To isolate coherent responses within separate frequency bands, we computed spatial coherence using a method of space-frequency singular value decomposition (SVD) [12]. The LFP signal was transformed from the time domain to the frequency domain:
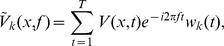
where 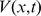 represents the LFP signal on channel 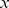 at time 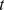, 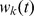 is the 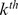 Slepian function at time 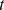 and bandwidth 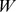. To decompose the signals, we performed an SVD on the complex matrix 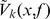:
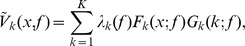
where 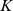 = 5. The coherence for a given frequency was measured by:
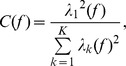
where 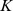 is the number of non-zero singular values. If the spatial pattern of activity is completely coherent in the given frequency band, there is only one non-zero singular value. Thus, 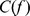 approaches 1 for highly coherent responses and 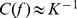 for random responses that are spatially uniform.

#### Filtering, Hilbert Transform, and Estimation of Wave Parameters

We applied a Kaiser filter [33] to band-pass-filter the LFP signals evoked by contrast reversal gratings. The passband of the Kaiser filter was 2–6, 4–8, 6–10, and 10–12 Hz for the responses evoked by gratings that contrast-reversed at 2, 3, 4, and 5 Hz, respectively. Other parameters of the Kaiser filter were: transition bandwidth = 1 Hz, passband ripple = 0.01 dB, and stopband attenuation = 60 dB [33]. Forward and backward filtering was used to prevent phase distortion. For the experiments in which we measured both the spontaneous responses and the evoked responses, spontaneous responses were filtered in a similar manner. After filtering, the signals in each channel were independently Z scored [18]. We used the Hilbert transform to extract the analytic phase from the band-limited signals 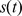 [17]. The analytical signal of 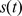 is defined as:
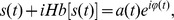
in which 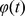 is the analytic phase, which is given by 
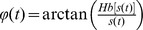
, and 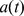 is the analytic amplitude. Although the analytic phase does not have a clear physiological meaning, the phases across space reflect the relative timing of activity across space [17].

For the filtered signal at each time 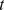 and coordinate 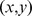 of the array, we calculated an instantaneous phase 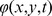. The phases in the array were unwrapped in both spatial and temporal dimensions, either for the purpose of computing phase gradient or displaying the phase map averaged over the time points within one cycle of oscillation [17]. The velocity of coherent activity was defined as the velocity of the contours of constant phase [34]. We computed the wave velocity, 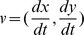, by taking the total derivative of 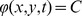 with respect to time [17]:
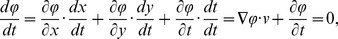
where 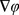 denotes the spatial gradient of the instantaneous phases across the array. To accurately estimate the speed and direction of the waves, for each time point we first defined a measure of phase gradient directionality (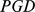) to determine the degree of alignment of the phase gradients across the array [17]:
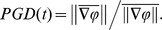
For a time point at which the phase gradients at all positions are perfectly aligned, 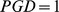. For a time point with randomly distributed phase gradients, 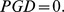 Since 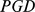 measures how well the phase gradients at all sites are aligned, responses at those time points with 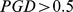 had well defined propagation velocity and direction [17], and thus were considered to be wave-like. To estimate the proportion of wave-like time points for the single-trial responses, we defined wave probability as the proportion of time points at which 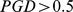. From the single-trial responses, we estimated the direction and speed of the waves using those time points with 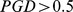. The wave direction was estimated by:
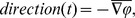
and the wave speed by:
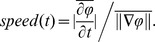



#### Linear Fit between Wave Speed and Oscillation Frequency

The relationship between wave speed and the frequency of neuronal oscillation was fitted by a linear function, 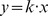. The goodness of fit was estimated by the 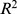 statistics:
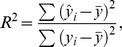
where 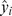 is the fitted value, 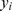 is the observed value, and 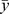 is the average of all observed values. An 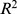 close to 1 indicates that the linear function well fits the data, whereas an 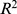 close to 0 indicates that the fit is not significantly better than approximating the data by its mean.

#### Wave Snapshots Display

To display the spatiotemporal pattern of the activity, the responses from each channel were normalized to their maximum amplitude [35]. The normalized signals were then color coded according to a linear pseudocolor scale. Data of broken channels were interpolated (Matlab function ‘interp2’). Each individual snapshot was again linearly interpolated for display purposes.

#### Fourier Analysis

For the evoked responses (i.e., the responses averaged over all trials) in each experiment, we also used Fourier analysis to obtain a phase map and analyze the phase gradient. To compute a single Fourier component for the evoked responses, we multiplied the raw signal 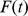 by the corresponding complex exponential:
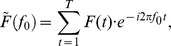
where 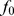 is the frequency of interest. The phase of this Fourier component is given by
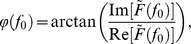
where 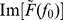 and 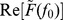 denote the imaginary and real parts of 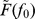, respectively. We then obtained a phase map using the phase of the 2^nd^ harmonic component in each channel. The phase obtained by this method represented the latency of response [10]. The gradient directionality (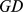) of the phase map was measured by 
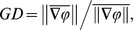
 where 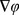 denotes the spatial gradient of the phases.

#### RF Mapping and RF Latency Estimation

Spatiotemporal RF maps were obtained by cross-correlating the LFP with the visual stimulus [36], [37]:
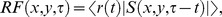
where 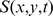 represents the spatiotemporal visual stimulus and 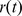 represents the LFP signal from a recording site. Since previous studies reported that the signal-to-noise ratio of responses to black squares was higher than that to white squares [38], [39] (which was confirmed by our own data), we used the RF mapped with black squares for subsequent analysis.

We computed the temporal impulse response as the variances of spatial RFs at different time delays [37], [40]:
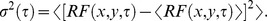
Spatial RF at peak variance was obtained for each recording site in the array. The location of the 17/18 border can be estimated from the changes in RF size and the reversal of RF progression [41], [42] (Figure S1). RF latency was defined as the time when the rising phase of the impulse response reached 40% of its peak value [43]. For each experiment, we measured the 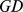 of RF latency map (a map that contained the latencies of all recording sites) by 
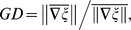
where 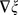 denotes the spatial gradient of the RF latencies across the array.

## Results

### Stimulus-Induced Oscillations Propagated As Waves Across the 17/18 Border

**Figure 1:**
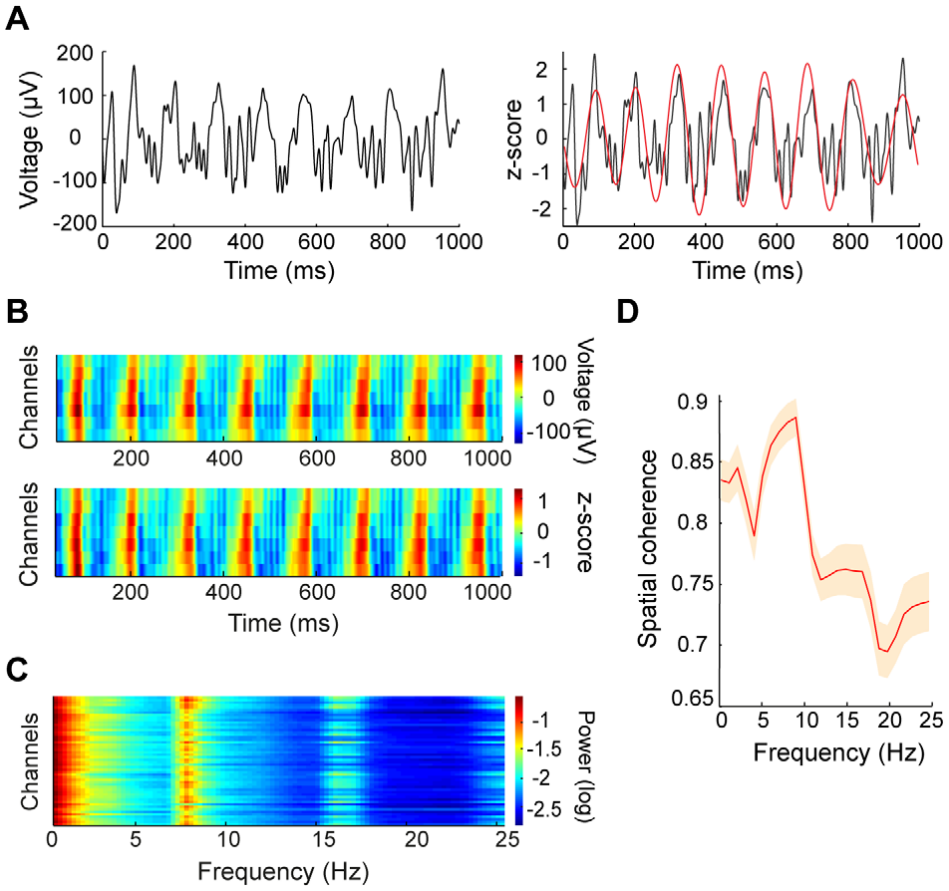
LFP responses to contrast reversal gratings and frequency analysis of the responses. (A) Left panel, raw LFP signal from a single channel during a single trial of visual stimulation by 4-Hz contrast reversal grating. Right panel, the black curve is the z-score signal for the trace shown in the left panel, and the red curve is the signal band-pass filtered at 6–10 Hz (2^nd^ harmonic response). (B) Upper panel, unfiltered LFP signals averaged over all trials in response to a 4-Hz contrast reversal grating (orientation = 135°) for 7 recording sites distributed along the medial-lateral axis. Lower panel, the z-score signal for the responses shown in the upper panel. (C) Power spectra for responses to 4-Hz contrast reversal gratings in all usable channels of the array from one experiment. Log power is presented. (D) Spatial coherence as a function of frequency computed from a space-frequency SVD analysis, averaged over 10 experiments. Shaded region: standard error of the mean.

**Figure 2:**
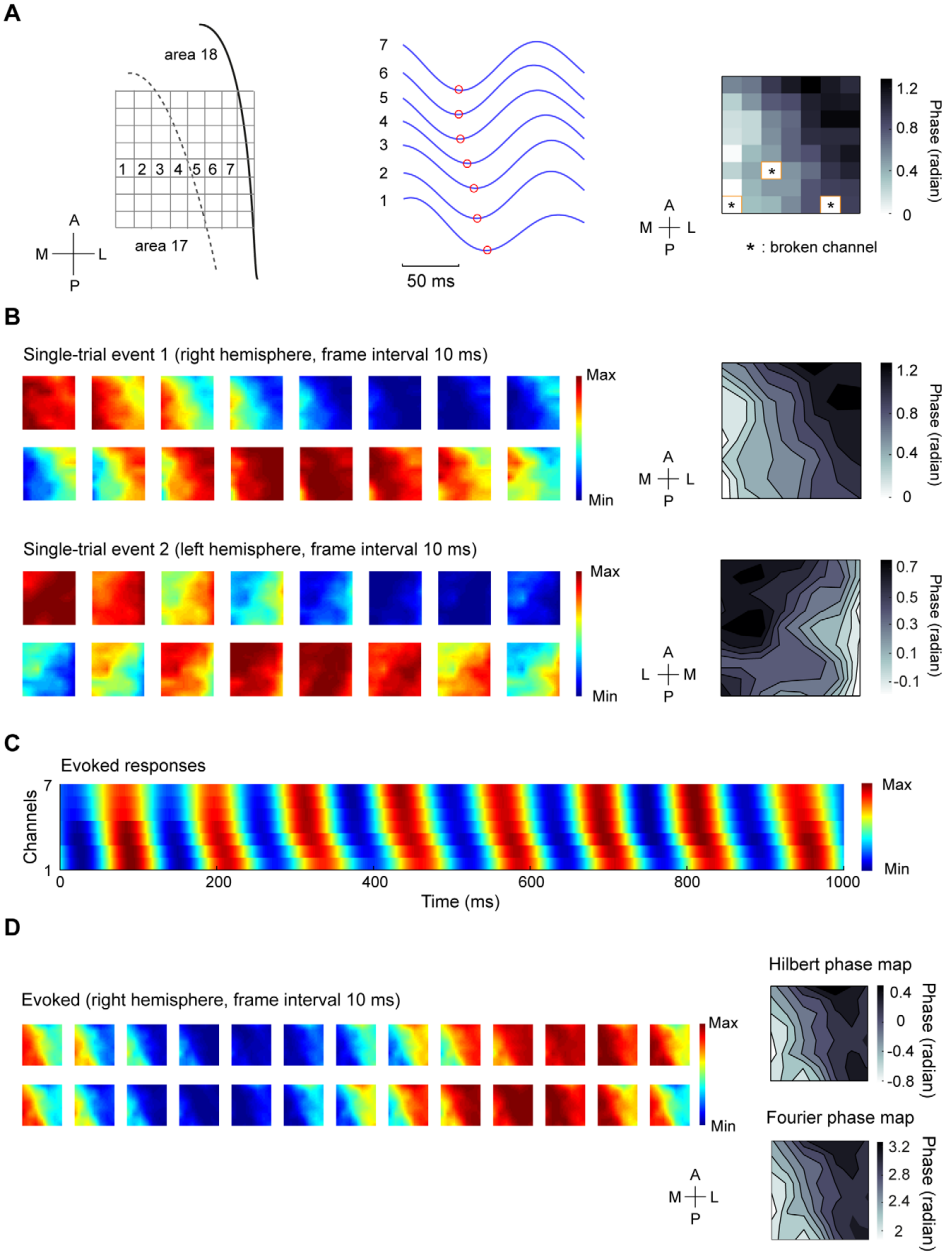
Waves in single-trial responses and evoked responses to 4-Hz contrast reversal gratings. (A) Left, schematic drawing of areas 17 and 18 (right hemisphere) and the recording sites of the 8×8 array. A, anterior; P, posterior; L, lateral; M, medial. Middle, Single-trial LFPs band-pass filtered at 6–10 Hz during one cycle of oscillation for the 7 recording sites indicated in the left panel. Each red circle marks the trough of response in each site. Right, phase map obtained by averaging the instantaneous phases over the time points within one cycle of oscillation shown in the middle panel for each recording site across the array. Broken channels were marked with ‘*’. Since the electrodes on the rightmost column of the array were not properly inserted into the cortex because the electrodes were near the lateral sulcus, only 8×7 of the recording sites were used for the analysis. (B) Left, time snapshots of two wave-like events from single-trial responses. The data for the first event was the same as those shown in (A). Right, two phase maps for the two wave-like events, respectively. The phase data were interpolated for display purpose. (C) Responses averaged over all trials (i.e., evoked responses) for the 7 recording sites indicated in the left panel of (A). Systematic phase shift can be observed in every cycle of the responses. (D) Left, snapshots of 2 cycles of evoked responses measured from one experiment in the right hemisphere. Upper right, a phase map computed by the method of Hilbert transform, Lower right, a phase map containing the phase of 2^nd^ harmonic component computed by Fourier analysis. The two phase maps were computed from the same data set. Data were interpolated for display purpose.

We recorded LFPs in response to full-field contrast reversal gratings from the superficial layers of areas 17 and 18 using multielectrode arrays (Materials and Methods). As shown in Figure 1A and B, 4-Hz contrast reversal gratings induced responses that oscillated at approximately 8 cycles/sec ( i.e., the 2^nd^ harmonic), consistent with previous studies [10], [44]. Power spectra of the LFPs across multiple channels showed prominent peaks at the 2^nd^ harmonic frequency (Figure 1C). To examine whether the broadband responses contained a distinct spatiotemporal structure of the 2^nd^ harmonic responses, we performed a space-frequency SVD analysis to compute the spatial coherence within different frequency bands (Materials and Methods). We found that the coherence peaked at frequencies around 8 Hz (Figure 1D). Because high spatial coherence is a necessary (but not sufficient) condition for wave-like activity [17], this result suggests that visually evoked LFPs may propagate as waves. We next extracted the visually induced oscillatory signals by band-pass filtering the responses at 6–10 Hz (Materials and Methods). During the oscillations in single trials, we observed systematic phase differences of the responses across different channels distributed along the medial-lateral axis (Figure 2A, middle). When we examined the spatial pattern of the normalized responses at a series of time points (Materials and Methods), we found plane waves traveling across the array (Figure 2B). To further examine the spatial organization of the phases, we extracted the instantaneous phase at each time point for each channel in the array (Materials and Methods). A phase map across the array was computed by averaging the phases over one cycle of oscillation for each channel (Figure 2A, right). For the wave-like responses shown in Figure 2B, the phase maps exhibited clear gradients, the directions of which were approximately along the medial-lateral axis (Figure 2B, right).

We further examined the responses averaged over all trials (i.e., evoked responses). Figure 2C shows the evoked responses for 7 recording sites across the array as indicated in Figure 2A. The presence of phase offsets in these sites suggests that, the wave activity is evoked by the stimulus rather than caused by random noise that would be cancelled out by averaging. Moreover, systematic phase shifts occurred in every cycle of the evoked responses, indicating that the propagating activity is in the form of one-cycle-one-wave [45]. Figure 2D shows the images of evoked responses for the time points within two cycles of oscillation recorded from the right hemisphere of one cat. In all 10 experiments, the propagation direction of the evoked waves was from area 18 to 17. To analyze the phase organization of the evoked responses shown in Figure 2D, we extracted the analytic phase at each time point using Hilbert transform (Materials and Methods) and computed a phase map by averaging the phases over all cycles of oscillation. We also performed Fourier analysis on the unfiltered LFPs and computed a phase map from the 2^nd^ harmonic component of the response (Materials and Methods). For the two phase maps estimated by the two methods (Figure 2D, right panel), the degree of phase shift across space was similar , with a systematic increase in phase from the medial to the lateral part of the cortex. For all 10 experiments, when we quantified the gradient of the 2^nd^ harmonic phase map by the measure of 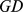 (Materials and Methods),we found that the GD was above 0.5 in most experiments (see below), further supporting the idea that the evoked responses propagated as waves.

### Direction and Speed of Stimulus-Induced Waves

**Figure 3:**
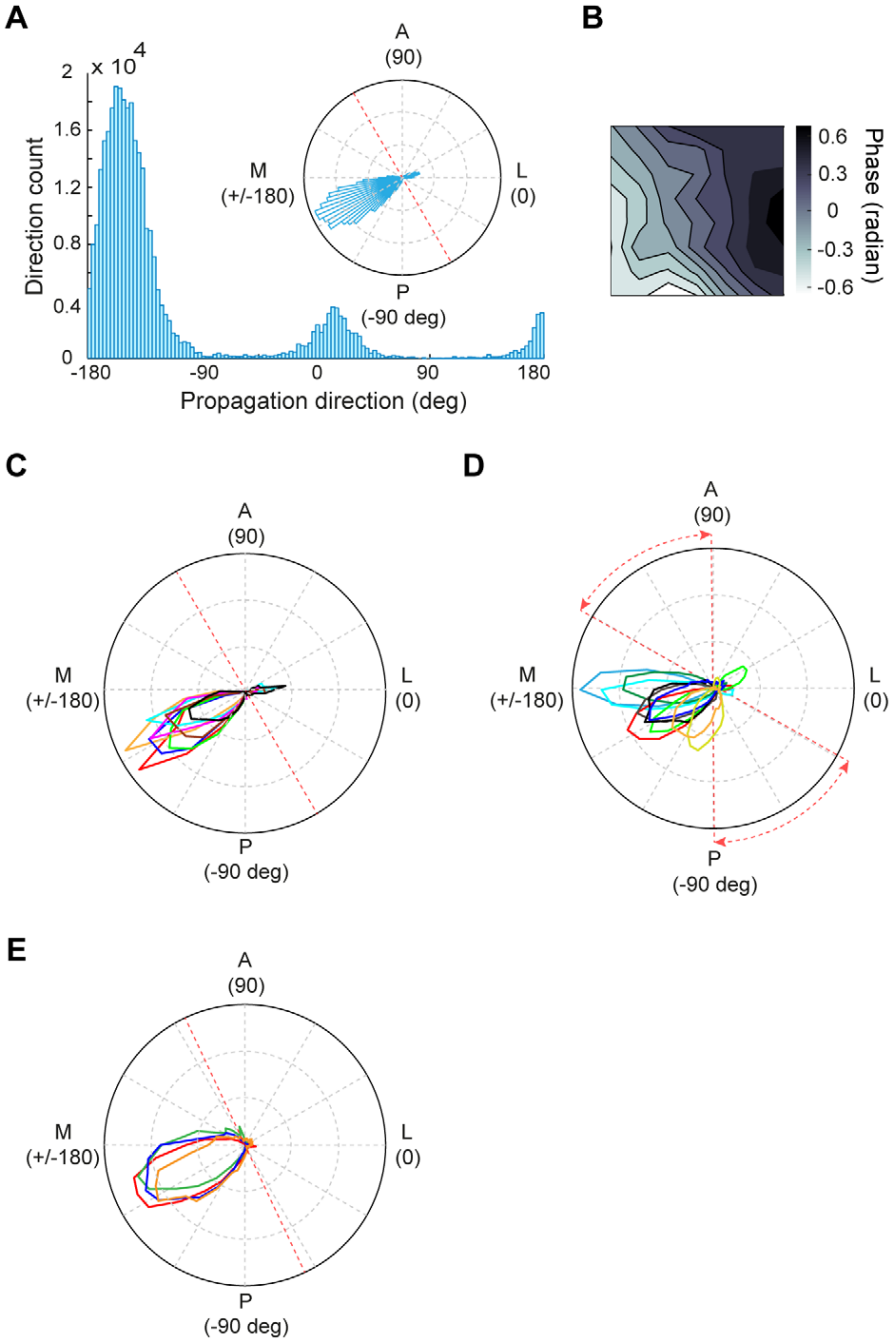
Analysis of wave direction for stimulus-induced responses. (A) Distribution of wave directions during all trials for the responses to 4-Hz contrast reversal gratings from one experiment. Red dashed line in the inset represents the 17/18 border. (B) Average phase map using the data points within 30° of the peak direction shown in (A). (C) Distribution of wave directions measured with 4-Hz contrast reversal gratings at different orientations from one experiment. Each color represents one orientation. Data were from the same experiment as that shown in (A). (D) Distribution of wave directions measured with 4-Hz contrast reversal gratings for all 10 experiments. Red dashed lines represent the range of the 17/18 border across different experiments. (E) Distribution of wave directions measured with gratings that contrast-reversed at 2, 3, 4, and 5 Hz, respectively, from one experiment. Red dashed line represents the 17/18 border.

**Figure 4:**
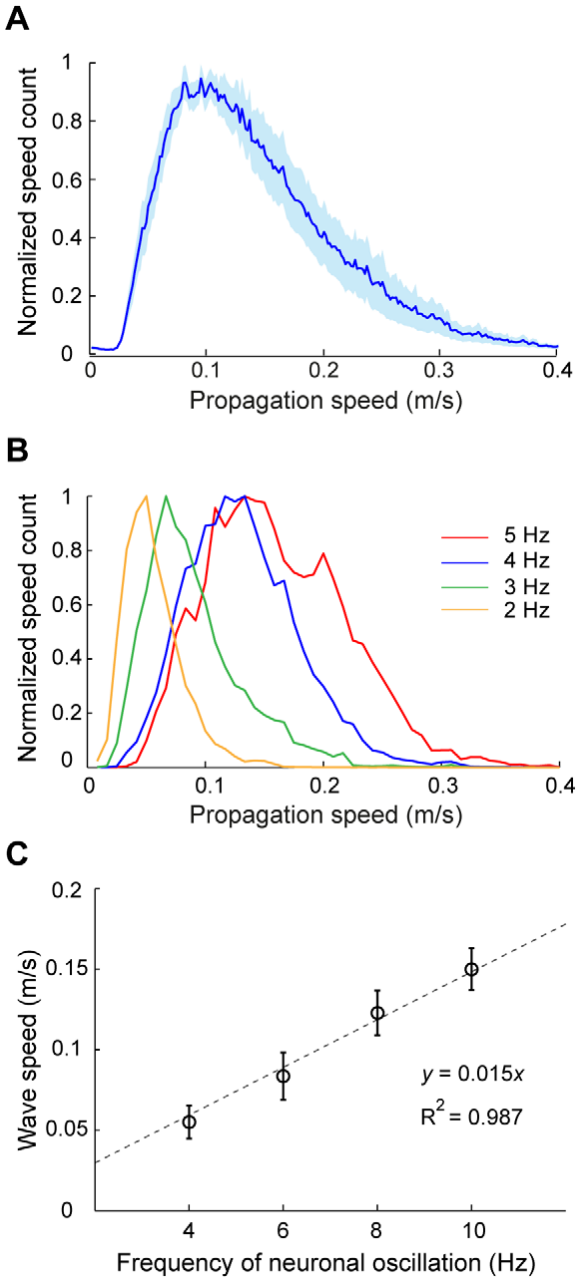
Analysis of wave speed for stimulus-induced responses. (A) Distribution of wave speeds for the responses to 4-Hz contrast reversal gratings for all 10 experiments. Shaded region: standard error of the mean. (B) Distributions of wave speeds for the responses to gratings that contrast reversed at 2, 3, 4, and 5 Hz, respectively, from one experiment. (C) Mean wave speed (mean ± SEM, n = 4) versus the frequency of neuronal oscillation, measured with gratings that contrast reversed at 2, 3, 4, or 5 Hz. The dashed line represents the linear fit (*y* = 0.015·*x*, R^2^ = 0.987).

To quantitatively characterize the waves in single trials, for each time point we used the analytic phases at all sites to obtain an instantaneous phase map, from which we further computed the instantaneous propagation direction and propagation speed (Materials and Methods). Because only a well-defined phase gradient indicates the presence of wave, we first quantified the degree of alignment of the phase gradients using the 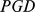 measure (Materials and Methods). 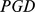 is 1 for a time point at which the phase gradients at all positions are perfectly aligned, i.e., the propagation direction is spatially coherent, and 0 if the phase gradients are random. We then used the time points at which the instantaneous 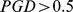 to estimate the wave direction (Materials and Methods). Figure 3A shows the distribution of propagating directions for waves recorded from one experiment. Clearly, the waves propagated in a dominant direction along the axis that was approximately parallel to the medial-lateral axis and almost perpendicular to the 17/18 border. To illustrate the spatial dynamics of this dominant propagation direction, we used the data points within 30° of the peak direction to plot an average phase map (Figure 3B), which also showed phase gradient across the array. For the stimulation condition of different orientations, the distributions of wave directions were similar (an example was shown in Figure 3C), suggesting that the waves may be mediated by intrinsic connectivity. In all 10 experiments, the dominant wave direction for single-trial responses was from area 18 to area 17 (Figure 3D). For those experiments in which we measured responses using gratings contrast-reversed at different temporal frequencies (2, 3, 4, or 5 Hz), the distributions of wave directions were also similar for different frequencies (an example was shown in Figure 3E). We also estimated the propagation speed using those time points with 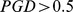. For the responses evoked by 4-Hz contrast reversal gratings (Figure 4A), the waves propagated with a mean speed of 0.12±0.03 m/sec (mean ± SD). When we examined the wave speed for responses evoked by stimuli at different temporal frequencies (Figure 4B), we found that the mean wave speed was positively correlated with the frequency of neuronal oscillation (*y* = 0.015·*x*, 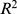 = 0.987, Figure 4C). Thus, the stimulus-evoked responses satisfied a dispersion relation [46], suggesting that the responses propagate as waves.

### RF Latency Cannot Account for the Phase Shifts of Evoked Responses

**Figure 5:**
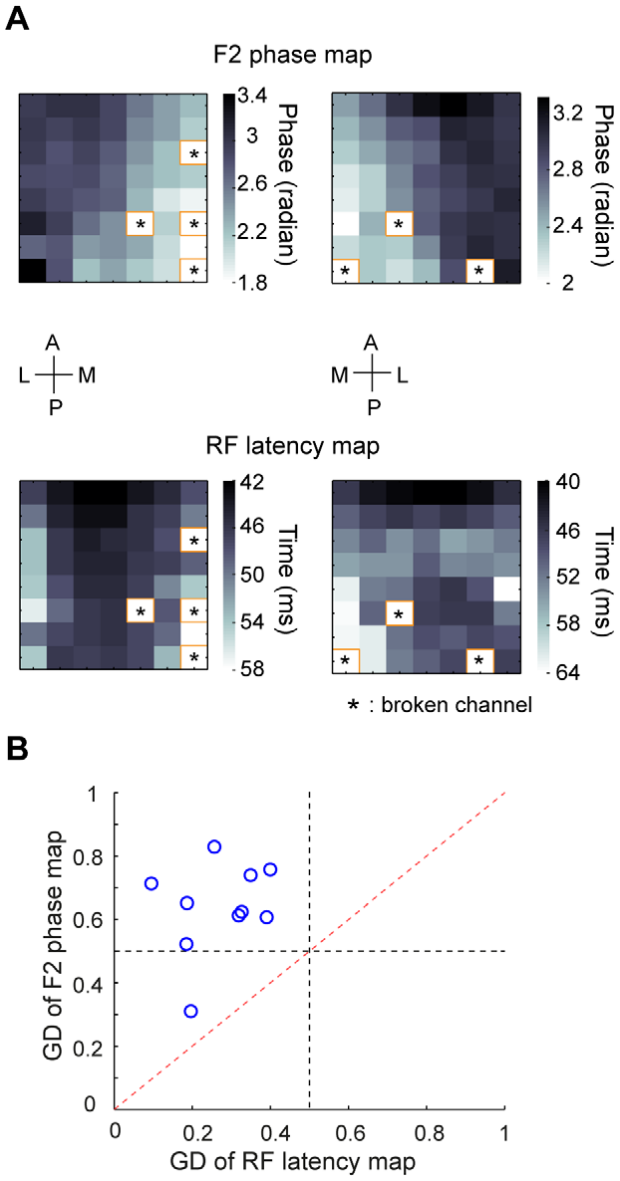
Comparison of the gradient between 2^nd^ harmonic phase map and RF latency map. (A) Upper, phase maps computed from the 2^nd^ harmonic responses of two experiments measured with 4-Hz contrast reversal gratings. Lower, RF latency maps measured in the same two experiments. Broken channels were marked with ‘*’. (B) Gradient directionality of the phase map was significantly larger than that of the RF latency map (P<0.01, n = 10, Wilcoxon signed rank test).

Theoretical studies suggest that traveling waves may arise from a network of weakly coupled oscillators [47]. If the waves we observed were explained by such a mechanism, the phase gradient would depend on the intra- and inter-cortical interactions. On the other hand, area 18 is known to be predominantly innervated by Y-type thalamic afferents, whereas area 17 is innervated by both X- and Y-type axons [48]. Since the conduction velocity of Y-type axons is faster than that of X-type axons [49], [50], the phase gradient across the 17/18 border may be explained by the spatial distribution of response latencies due to feedforward inputs. To examine this issue, we measured the LFPs in response to sparse noise stimuli, in which each small square was supposed to mainly activate the feedforward inputs. Response latency for each site was estimated from the temporal impulse response of the RF (Materials and Methods, Figure S1). As shown in Figure 5A, while the 2^nd^ harmonic phase maps measured with large stimuli exhibited a clear gradient along the medial-lateral axis, there was no such gradient in the RF latency maps. The 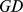 for the 2^nd^ harmonic phase map was significantly higher than that for the RF latency map (P<0.01, n = 10, Wilcoxon signed rank test, Figure 5B), suggesting that the systematic phase differences evoked by the full-field contrast reversal gratings cannot be fully accounted for by the timing differences of the feedforward inputs. Instead, intra- and inter-cortical interactions [47] may be involved in generating the visually evoked waves.

### Comparison Between Evoked Waves and Spontaneous Waves

**Figure 6:**
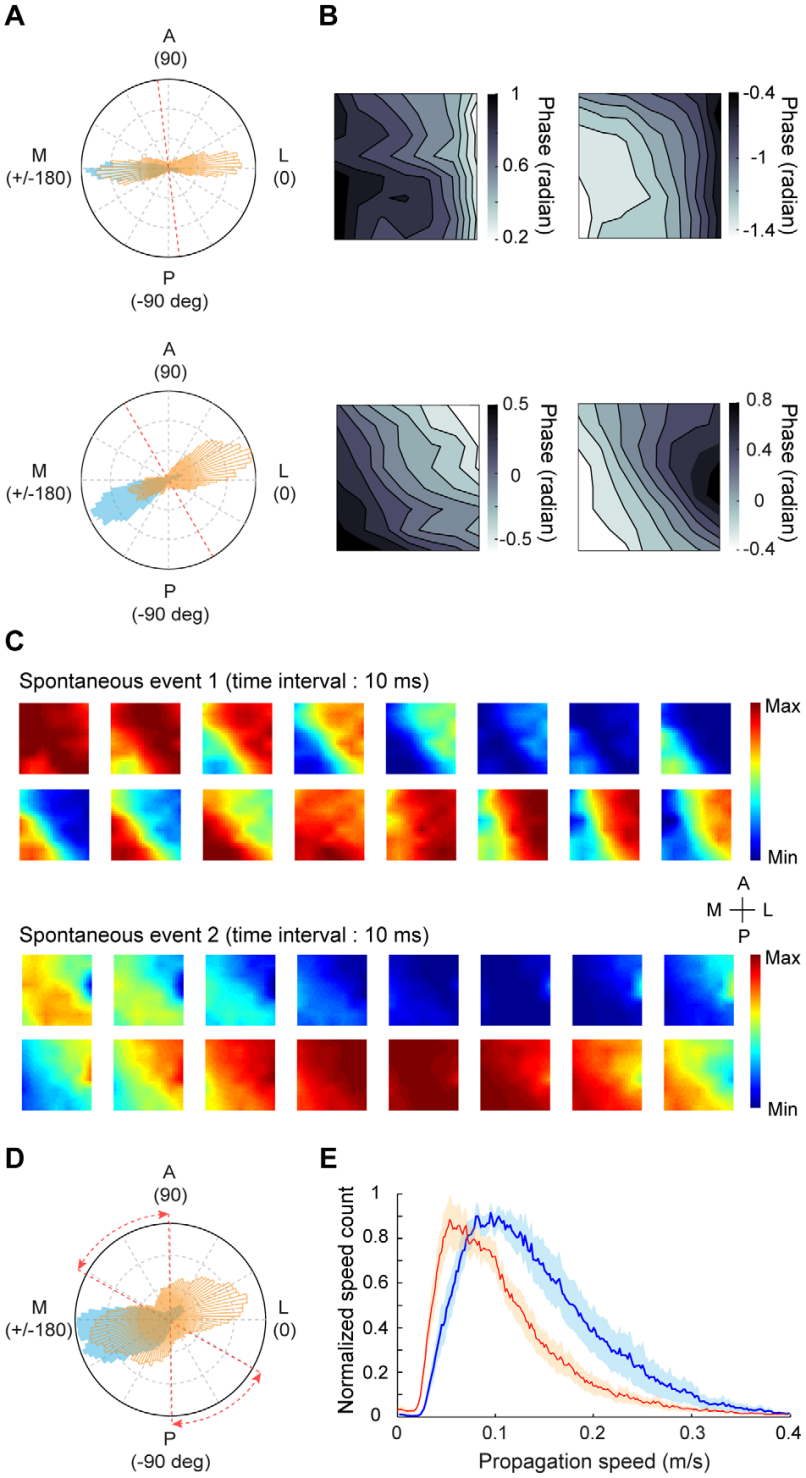
Comparison of wave parameters between spontaneous responses and responses induced by 4-Hz contrast reversal gratings. (A) Distributions of wave directions for stimulus-induced (blue) and spontaneous (orange) activities measured in two experiments. Red dashed line indicates the 17/18 border. (B) Upper, average phase maps computed using data points within 30° of the two peak directions for the spontaneous activity shown in the upper panel of (A). Lower, average phase maps computed using data points within 30° of the two peaks for the spontaneous activity shown in the lower panel of (A). (C) Snapshots of two example spontaneous events recorded from the same experiment. (D) Distribution of wave directions for stimulus-induced (blue) and spontaneous (orange) activities, averaged from 9 experiments. Red dashed lines represent the range of the 17/18 border across different experiments. (E) Distribution of wave speeds for stimulus-induced (blue) and spontaneous (red) activities for 9 experiments. Shaded region: standard error of the mean.

Previous studies showed that spontaneous responses also propagated as waves [9], [16], and the spatiotemporal patterns of spontaneous activity resembled the sensory-evoked responses [51]. Since the visually evoked waves were likely mediated by intrinsic cortical connections, we wondered whether a similar wave pattern was present in the spontaneous activity. We compared the spontaneous responses and the responses evoked by 4-Hz contrast reversal gratings measured from the same animals (Materials and Methods). We analyzed the spatial coherence of spontaneous responses across all frequency bands and found that high coherence was limited to low frequencies (Figure S2A). Because our method to characterize wave parameters only applies to signals in a narrow frequency band (Materials and Methods), we band-pass filtered the spontaneous responses at 6–10 Hz, in order to compare them with the 8-Hz oscillatory responses evoked by the 4-Hz contrast reversal gratings. Similar to the stimulus-evoked responses, we obtained the analytic phase by the method of Hilbert transform and analyzed the phase gradient for each time point of the spontaneous responses. We first compared the wave probability (Materials and Methods) between the evoked and spontaneous activities. We found that wave probability was significantly higher in the stimulus-evoked (27.0%±1.6%, mean ± SEM) than the spontaneous activity (17.7%±1.9%, mean ± SEM) (P<0.05, Wilcoxon signed rank test). We then estimated wave direction and wave speed for spontaneous activity using those time points at which 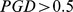. Figure 6A shows the distributions of wave directions for spontaneous (orange) and evoked activities (blue) from two experiments. In contrast with the evoked waves, the spontaneous waves appeared to show two propagating directions. For the spontaneous activity, average phase maps computed using data points within 30° of the two peak directions showed opposite phase gradients (Figure 6B). Snapshots of different spontaneous events measured in the same animal also revealed opposite wave directions (Figure 6C). In all 9 experiments, we found that the spontaneous activity propagated with two major directions, either from area 17 to 18 or from area 18 to 17, whereas the evoked activity mostly propagated from area 18 to 17 (Figure 6D). When we analyzed the spontaneous responses at a broader frequency band (3–10 Hz), we also observed two peaks in the distribution of wave directions (Figure S2B). This suggests that visual stimulation modulate the propagating directions of ongoing wave activity. In addition to the difference in propagating direction, the distribution of wave speed was significantly shifted to higher values under visual stimulation than spontaneous condition (P<0.005, n = 9, Kolmogorov-Smirnov test, Figure 6E). Such speed difference between evoked and spontaneous responses increased with the frequency of neuronal oscillation (Figure S2C).

## Discussion

We have investigated the spatiotemporal pattern of population activity in cat visual cortex using LFP recordings with multielectrode arrays. We found that the stimulus-entrained oscillatory activity propagated as waves from area 18 to 17. The direction of the evoked waves was not affected by the stimulus orientation and could not be predicted from the spatial distribution of RF latencies measured with local stimuli. Propagating waves were also observed in the spontaneous activity, which were bidirectional between areas 17 and 18. Furthermore, wave probability and wave speed were higher in the stimulus-induced than the spontaneous responses. Together, these results suggest that visual stimulation may modulate the spontaneous activity to facilitate the information flow from area 18 to 17.

Anatomical and physiological studies show that areas 17 and 18 receive direct inputs from LGN and the two areas process different aspects of visual information in a parallel manner [52]. Area 17 is mainly activated by X- and Y-fibers from LGN, whereas area 18 is activated by the Y-signals [48]. In addition to receiving ascending LGN inputs, areas 17 and 18 also receive cortico-cortical inputs from each other [23], [24], [25]. When the Y inputs from LGN were deactivated, neurons in area 18 were tuned to slower stimuli, suggesting that area 17 may activate area 18 through cortico-cortical connections [53]. By inactivating the activity in LGN, Mignard and Malpeli [26] showed that the cells in layers 2/3 of area 17 were well driven if area 18 was intact, but their responses were reduced when area 18 was destroyed, suggesting that neurons in layers 2/3 of area 18 can directly drive neurons in layers 2/3 of area 17. In agreement with this result, Martinez-Conde et al [54] showed that pharmacological blockade of neuronal activity in layers 2/3 of area 18 could change the visual responses of cells in layers 2/3 of area 17. In our study, we found that the spontaneous activity propagated as traveling waves along both directions between areas 17 and 18, which may be mediated by the intrinsic cortico-cortical connections. During oscillatory responses elicited by visual stimulation, the waves predominantly traveled from area 18 to 17, suggesting that cortical processing of visual information involves modulating the spatiotemporal patterns of spontaneous activity. To determine whether the direction of evoked waves is due to the latency difference inherit from the feedforward inputs, we measured the latencies for RFs in all recording sites. The spatial distribution of RF latencies did not exhibit clear gradient, suggesting that the evoked waves may be largely mediated by intrinsic cortical circuits and may emerge from the modulation of spontaneous activity.

We used wave probability, defined as the proportion of time points at which 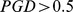, to estimate the proportion of wave-like time points for single-trial responses. The low percentage of wave-like time points found in our study may be due to the following reasons. First, the visually-evoked waves may be suppressed by the spontaneous activity, as demonstrated by previous studies [28], [55]. Second, we detected waves based on the gradients of phase maps, which had low spatial resolution (8×7 resolution in most cases) and thus may be sensitive to noise. For example, if one channel was contaminated by noise, phase gradients at the neighbouring four channels would be inaccurate. This may decrease the degree of alignment among the phase gradients across the array, leading to a decrease in the proportion of time points with 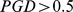. Although the wave probability in single trials was low, waves in the evoked responses were continuous and propagated in a pattern of one-cycle-one wave. We analyzed the probability of time points with 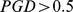 for each cycle of the single-trial responses induced by 4-Hz contrast reversal gratings (Figure S3). Although the probability in the first and the last cycle was a bit lower than that in the other cycles (probably because the stimulus onset and offset disrupted the neuronal oscillation induced by the contrast reversal gratings), the probability was more or less close to 27% in each cycle. Since the wave-like activity was present in each cycle, the pattern of one-cycle-one-wave could be preserved by averaging over trials. Generally, we found that evoked responses could propagate as waves as long as the responses were well entrained by the contrast reversal gratings.

Using voltage-sensitive dye (VSD) imaging in rat visual cortex, Xu et al [9] reported that the visually evoked waves were initiated in V1 and were compressed/reflected at the V1/V2 border, whereas the spontaneous waves propagated across the cortex without compression and reflection. Although our method could not analyze compression/reflection of the waves, both our study and this previous work showed that the evoked waves exhibited differences from the spontaneous waves. Using VSD imaging in cat visual cortex, Benucci et al [10] examined waves in the space domain and orientation domain, respectively, with focal or full-field contrast reversal gratings. In their analysis of the cortical responses to full-field stimuli, the authors focused more on the phase differences across different orientation columns than those across different cortical distances. While Benucci et al [10] investigated the difference between circuits that underlie spatial selectivity and orientation selectivity, our study aimed to explore the spatiotemporal pattern of population activity across cortical areas. By imaging cortical responses to drifting gratings, a recent study by Onat et al [56] also observed propagating waves in areas 17 and 18, but the wave direction was dependent on the drifting direction of the grating and the wave speed was related to the speed of the grating.

Wave activity has mostly been studied using VSD imaging, which revealed a variety of wave patterns, including plane waves, spiral waves, and target waves [45]. Quantitative description of the waves is essential for us to understand the dynamics of population activity. Early work used a method of SVD to identify wave patterns [12]. However, it is difficult to use this method to automatically detect waves. The algorithm we adapted was based on computing the gradient of instantaneous phase obtained by Hilbert transform [17]. This method allows us to describe propagating activity in the oscillations in a specified frequency band, estimate wave direction and wave speed from single-trial responses, and detect waves under both evoked and spontaneous conditions. However, the method we used can only be applied to plane waves. Recent work described an algorithm based on comparing the correlation of temporal features across space with established flow templates [22], [57]. This method works well in detecting target waves and spiral waves in addition to plane waves, but the method is computationally intensive.

The functional significance of the waves is still unclear. Since the direction of evoked waves was independent of stimulus orientation, we speculate that the waves may be involved in communication between areas 17 and 18 rather than coding of specific visual features. Previous studies in cat visual cortex suggested that the interaction between areas 17 and 18 plays an important role in the integration of border and surface information [58], [59]. In addition, neurons in areas 17 and 18 can respond to second-order stimuli [60], [61], which may be important in figure-ground segregation. It is of interest for future study to use more complex stimuli to investigate whether the waves across areas 17 and 18 contribute to visual scene segmentation. Furthermore, phase information of neuronal oscillations is important in cortical processing. For example, the spike times of a single neuron relative to the phase of network oscillation can be used to carry stimulus information [33]. Future studies may explore the functions of waves by combining information coding and the spatiotemporal pattern of oscillation phases.

## Supporting Information

Figure S1
**RF measurement for all sites across the array.** (A) Upper, variance of spatial RF map as a function of time after stimulus onset for one recording site. Red dashed line marks the RF latency. Lower, spatial RF maps at different time delays. (B) Spatial RF map at peak variance for each recording site in the array. A, anterior; P, posterior; L, lateral; M, medial. We fitted each RF with a two-dimensional Gaussian, 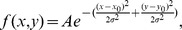 where 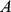, 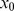, 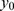, and 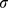 are free parameters. The location of the 17/18 border can be estimated from the changes in RF size and the reversal of RF progression. Red arrow in each row points to the site at which the reversal of RF progression occurred. In the RF maps along the row pointed by a black arrow, each circle represents the contour of Gaussian fit at 1 SD. (C) Gaussian fits of the RFs are shown for the 7 recording sites in the row marked by a black arrow in (B).(TIF)

Figure S2 **Analysis of spontaneous responses.** (A) Spatial coherence as a function of frequency. Shaded region: standard error of the mean. (B) Distribution of wave directions for spontaneous responses band-pass filtered at 3–10 Hz, averaged from 9 experiments. Red dashed lines indicate the range of 17/18 border. (C) Comparison of wave speed between spontaneous and stimulus-induced responses at different oscillation frequencies. Red circles: mean wave speed for the spontaneous responses band-pass filtered at 2–6, 4–8, 6–10, and 10–12 Hz, respectively. The red dashed line represents the linear fit (*y* = 0.012·*x*, R^2^ = 0.991). Blue circles, mean wave speed for the stimulus-induced responses that oscillated at 4, 6, 8, 10 Hz, respectively. The blue dashed line represents the linear fit (*y* = 0.015·*x*, R^2^ = 0.987). Error bars are SEM. Results were from 4 experiments.
(TIF)

Figure S3
**Wave probability for each cycle of the evoked responses.** Proportion of time points with 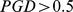 was computed for the single-trial responses in each cycle of neuronal oscillation induced by 4-Hz contrast reversal gratings (n = 10).(TIF)

## References

[1] Kuramoto Y (1984).

[2] Kopell N, Ermentrout GB (1986). Symmetry and phaselocking in chains of weakly coupled oscillators.. Communications on Pure and Applied Mathematics.

[3] Breakspear M, Heitmann S, Daffertshofer A (2010). Generative models of cortical oscillations: neurobiological implications of the kuramoto model.. Front Hum Neurosci.

[4] Orbach HS, Cohen LB (1983). Optical monitoring of activity from many areas of the in vitro and in vivo salamander olfactory bulb: a new method for studying functional organization in the vertebrate central nervous system.. J Neurosci.

[5] Lam YW, Cohen LB, Wachowiak M, Zochowski MR (2000). Odors elicit three different oscillations in the turtle olfactory bulb.. J Neurosci.

[6] Delaney KR, Gelperin A, Fee MS, Flores JA, Gervais R (1994). Waves and stimulus-modulated dynamics in an oscillating olfactory network.. Proc Natl Acad Sci U S A.

[7] Lubenov EV, Siapas AG (2009). Hippocampal theta oscillations are travelling waves.. Nature.

[8] Grinvald A, Lieke EE, Frostig RD, Hildesheim R (1994). Cortical point-spread function and long-range lateral interactions revealed by real-time optical imaging of macaque monkey primary visual cortex.. J Neurosci.

[9] Xu W, Huang X, Takagaki K, Wu JY (2007). Compression and reflection of visually evoked cortical waves.. Neuron.

[10] Benucci A, Frazor RA, Carandini M (2007). Standing waves and traveling waves distinguish two circuits in visual cortex.. Neuron.

[11] Roland PE, Hanazawa A, Undeman C, Eriksson D, Tompa T (2006). Cortical feedback depolarization waves: a mechanism of top-down influence on early visual areas.. Proc Natl Acad Sci U S A.

[12] Prechtl JC, Cohen LB, Pesaran B, Mitra PP, Kleinfeld D (1997). Visual stimuli induce waves of electrical activity in turtle cortex.. Proc Natl Acad Sci U S A.

[13] Song WJ, Kawaguchi H, Totoki S, Inoue Y, Katura T (2006). Cortical intrinsic circuits can support activity propagation through an isofrequency strip of the guinea pig primary auditory cortex.. Cereb Cortex.

[14] Kleinfeld D, Delaney KR (1996). Distributed representation of vibrissa movement in the upper layers of somatosensory cortex revealed with voltage-sensitive dyes.. J Comp Neurol.

[15] Petersen CC, Grinvald A, Sakmann B (2003). Spatiotemporal dynamics of sensory responses in layer 2/3 of rat barrel cortex measured in vivo by voltage-sensitive dye imaging combined with whole-cell voltage recordings and neuron reconstructions.. J Neurosci.

[16] Han F, Caporale N, Dan Y (2008). Reverberation of recent visual experience in spontaneous cortical waves.. Neuron.

[17] Rubino D, Robbins KA, Hatsopoulos NG (2006). Propagating waves mediate information transfer in the motor cortex.. Nat Neurosci.

[18] Nauhaus I, Busse L, Carandini M, Ringach DL (2009). Stimulus contrast modulates functional connectivity in visual cortex.. Nat Neurosci.

[19] Gilbert CD, Wiesel TN (1983). Clustered intrinsic connections in cat visual cortex.. J Neurosci.

[20] Senseman DM, Robbins KA (1999). Modal behavior of cortical neural networks during visual processing.. J Neurosci.

[21] Senseman DM, Robbins KA (2002). High-speed VSD imaging of visually evoked cortical waves: decomposition into intra- and intercortical wave motions.. J Neurophysiol.

[22] Takagaki K, Zhang C, Wu JY, Lippert MT (2008). Crossmodal propagation of sensory-evoked and spontaneous activity in the rat neocortex.. Neuroscience Letters.

[23] Symonds LL, Rosenquist AC (1984). Corticocortical connections among visual areas in the cat.. J Comp Neurol.

[24] Symonds LL, Rosenquist AC (1984). Laminar origins of visual corticocortical connections in the cat.. J Comp Neurol.

[25] Salin PA, Kennedy H, Bullier J (1995). Spatial reciprocity of connections between areas 17 and 18 in the cat.. Can J Physiol Pharmacol.

[26] Mignard M, Malpeli JG (1991). Paths of information flow through visual cortex.. Science.

[27] Bullier J, McCourt ME, Henry GH (1988). Physiological studies on the feedback connection to the striate cortex from cortical areas 18 and 19 of the cat.. Exp Brain Res.

[28] Petersen CC, Hahn TT, Mehta M, Grinvald A, Sakmann B (2003). Interaction of sensory responses with spontaneous depolarization in layer 2/3 barrel cortex.. Proc Natl Acad Sci U S A.

[29] Wang C, Yao H (2011). Sensitivity of V1 neurons to direction of spectral motion.. Cereb Cortex.

[30] Movshon JA, Thompson ID, Tolhurst DJ (1978). Spatial and temporal contrast sensitivity of neurones in areas 17 and 18 of the cat's visual cortex.. J Physiol.

[31] DeAngelis GC, Ohzawa I, Freeman RD (1993). Spatiotemporal organization of simple-cell receptive fields in the cat's striate cortex. II. Linearity of temporal and spatial summation.. J Neurophysiol.

[32] Percival DB, Walden AT (1993). Spectral analysis for physical applications : multitaper and conventional univariate techniques.

[33] Montemurro MA, Rasch MJ, Murayama Y, Logothetis NK, Panzeri S (2008). Phase-of-firing coding of natural visual stimuli in primary visual cortex.. Curr Biol.

[34] Fleet DJ, Jepson AD (1990). Computation of Component Image Velocity from Local Phase Information.. International Journal of Computer Vision.

[35] Jin W, Zhang RJ, Wu JY (2002). Voltage-sensitive dye imaging of population neuronal activity in cortical tissue.. J Neurosci Methods.

[36] Jones JP, Palmer LA (1987). The two-dimensional spatial structure of simple receptive fields in cat striate cortex.. J Neurophysiol.

[37] Xing D, Yeh CI, Shapley RM (2009). Spatial spread of the local field potential and its laminar variation in visual cortex.. J Neurosci.

[38] Yeh CI, Xing D, Shapley RM (2009). “Black” responses dominate macaque primary visual cortex v1.. J Neurosci.

[39] Jin JZ, Weng C, Yeh CI, Gordon JA, Ruthazer ES (2008). On and off domains of geniculate afferents in cat primary visual cortex.. Nat Neurosci.

[40] Malone BJ, Kumar VR, Ringach DL (2007). Dynamics of receptive field size in primary visual cortex.. J Neurophysiol.

[41] Tusa RJ, Palmer LA, Rosenquist AC (1978). The retinotopic organization of area 17 (striate cortex) in the cat.. J Comp Neurol.

[42] Tusa RJ, Rosenquist AC, Palmer LA (1979). Retinotopic organization of areas 18 and 19 in the cat.. J Comp Neurol.

[43] Jin J, Wang Y, Lashgari R, Swadlow HA, Alonso JM (2011). Faster thalamocortical processing for dark than light visual targets.. J Neurosci.

[44] Movshon JA, Thompson ID, Tolhurst DJ (1978). Spatial summation in the receptive fields of simple cells in the cat's striate cortex.. J Physiol.

[45] Wu JY, Huang X, Zhang C (2008). Propagating Waves of Activity in the Neocortex: What They Are, What They Do.. Neuroscientist.

[46] Nunez PL, Srinivasan R (2006). A theoretical basis for standing and traveling brain waves measured with human EEG with implications for an integrated consciousness.. Clinical Neurophysiology.

[47] Ermentrout GB, Kleinfeld D (2001). Traveling electrical waves in cortex: insights from phase dynamics and speculation on a computational role.. Neuron.

[48] Dreher B, Leventhal AG, Hale PT (1980). Geniculate input to cat visual cortex: a comparison of area 19 with areas 17 and 18.. J Neurophysiol.

[49] Cleland BG, Dubin MW, Levick WR (1971). Sustained and transient neurones in the cat's retina and lateral geniculate nucleus.. J Physiol.

[50] Hoffmann KP, Stone J, Sherman SM (1972). Relay of receptive-field properties in dorsal lateral geniculate nucleus of the cat.. J Neurophysiol.

[51] Kenet T, Bibitchkov D, Tsodyks M, Grinvald A, Arieli A (2003). Spontaneously emerging cortical representations of visual attributes.. Nature.

[52] Payne BR, Peters A, Payne BR, Peters A (2002). The concept of cat primary visual cortex.. The cat primary visual cortex.

[53] Dreher B, Michalski A, Cleland BG, Burke W (1992). Effects of selective pressure block of Y-type optic nerve fibers on the receptive-field properties of neurons in area 18 of the visual cortex of the cat.. Vis Neurosci.

[54] Martinez-Conde S, Cudeiro J, Grieve KL, Rodriguez R, Rivadulla C (1999). Effects of feedback projections from area 18 layers 2/3 to area 17 layers 2/3 in the cat visual cortex.. J Neurophysiol.

[55] Arieli A, Sterkin A, Grinvald A, Aertsen A (1996). Dynamics of ongoing activity: explanation of the large variability in evoked cortical responses.. Science.

[56] Onat S, Nortmann N, Rekauzke S, Konig P, Jancke D (2011). Independent encoding of grating motion across stationary feature maps in primary visual cortex visualized with voltage-sensitive dye imaging.. Neuroimage.

[57] Takagaki K, Zhang C, Wu JY, Ohl FW (2011). Flow detection of propagating waves with temporospatial correlation of activity.. J Neurosci Methods.

[58] Hung CP, Ramsden BM, Chen LM, Roe AW (2001). Building surfaces from borders in Areas 17 and 18 of the cat.. Vision Res.

[59] Hung CP, Ramsden BM, Roe AW (2007). A functional circuitry for edge-induced brightness perception.. Nat Neurosci.

[60] Zhou YX, Baker CL (1996). Spatial properties of envelope-responsive cells in area 17 and 18 neurons of the cat.. J Neurophysiol.

[61] Baker CL (1999). Central neural mechanisms for detecting second-order motion.. Curr Opin Neurobiol.

