# Supplementary figures and images for: Stimulus-Entrained Oscillatory Activity Propagates as Waves from Area 18 to 17 in Cat Visual Cortex
Source: PLoS One. 2012 Jul 25;7(7):e41960. doi: 10.1371/journal.pone.0041960 (PMC3405032; doi:10.1371/journal.pone.0041960)

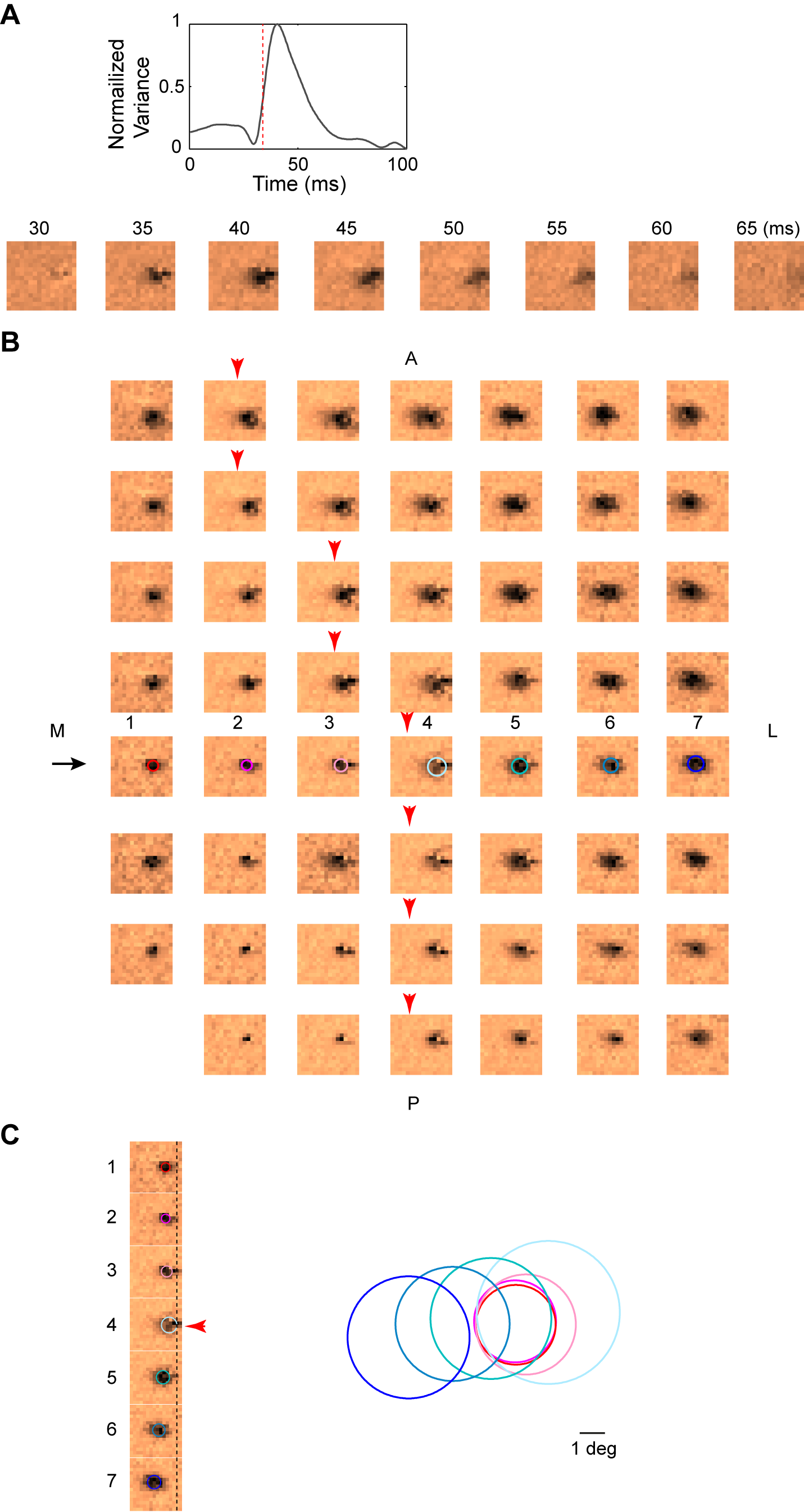

Supplement: Figure S1 — RF measurement for all sites across the array. (A) Upper, variance of spatial RF map as a function of time after stimulus onset for one recording site. Red dashed line marks the RF latency. Lower, spatial RF maps at different time delays. (B) Spatial RF map at peak variance for each recording site in the array. A, anterior; P, posterior; L, lateral; M, medial. We fitted each RF with a two-dimensional Gaussian, where , , , and are free parameters. The location of the 17/18 border can be estimated from the changes in RF size and the reversal of RF progression. Red arrow in each row points to the site at which the reversal of RF progression occurred. In the RF maps along the row pointed by a black arrow, each circle represents the contour of Gaussian fit at 1 SD. (C) Gaussian fits of the RFs are shown for the 7 recording sites in the row marked by a black arrow in (B). (TIF) [file pone.0041960.s001.tif]

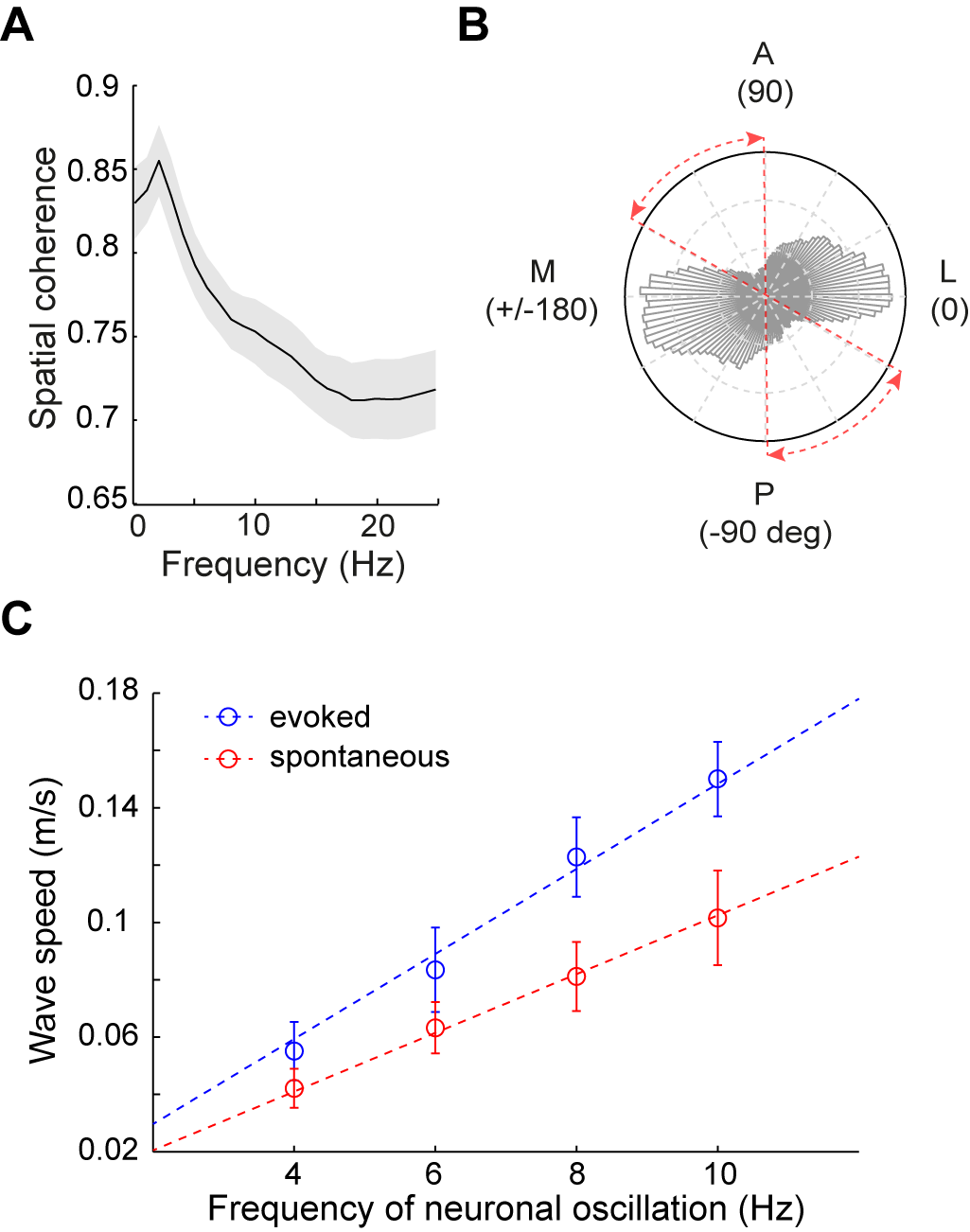

Supplement: Figure S2 — Analysis of spontaneous responses. (A) Spatial coherence as a function of frequency. Shaded region: standard error of the mean. (B) Distribution of wave directions for spontaneous responses band-pass filtered at 3–10 Hz, averaged from 9 experiments. Red dashed lines indicate the range of 17/18 border. (C) Comparison of wave speed between spontaneous and stimulus-induced responses at different oscillation frequencies. Red circles: mean wave speed for the spontaneous responses band-pass filtered at 2–6, 4–8, 6–10, and 10–12 Hz, respectively. The red dashed line represents the linear fit (y = 0.012·x, R2 = 0.991). Blue circles, mean wave speed for the stimulus-induced responses that oscillated at 4, 6, 8, 10 Hz, respectively. The blue dashed line represents the linear fit (y = 0.015·x, R2 = 0.987). Error bars are SEM. Results were from 4 experiments. (TIF) [file pone.0041960.s002.tif]

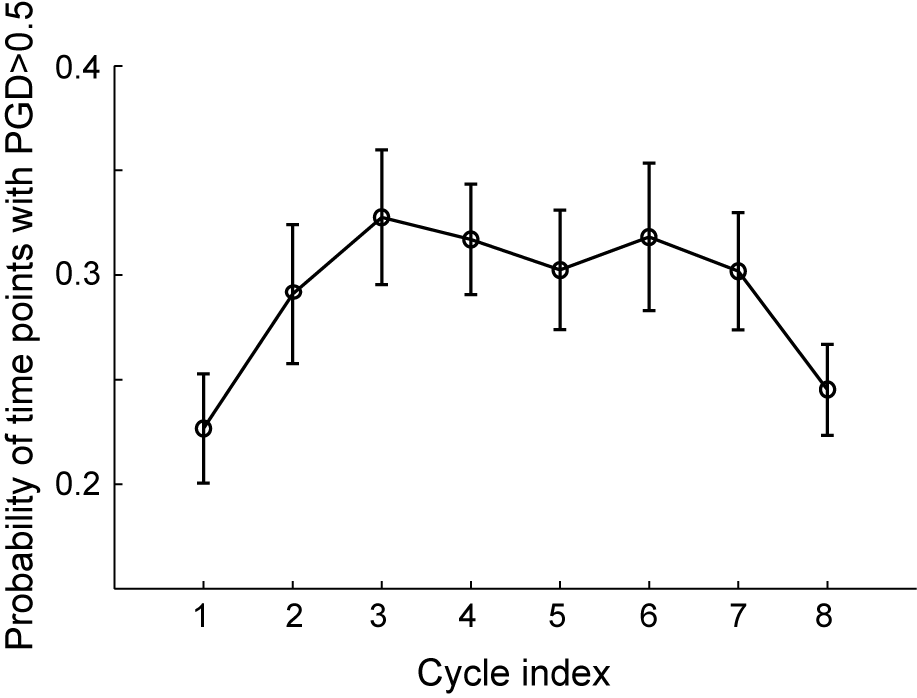

Supplement: Figure S3 — Wave probability for each cycle of the evoked responses. Proportion of time points with was computed for the single-trial responses in each cycle of neuronal oscillation induced by 4-Hz contrast reversal gratings (n = 10). (TIF) [file pone.0041960.s003.tif]
